# CASE REPORT A Double Thoracodorsal Artery Perforator Flap Technique for the Treatment of Pectus Excavatum

**Published:** 2010-04-30

**Authors:** Raphaël Sinna, David Perignon, Quentin Qassemyar, Thomas Benhaim, Codrin N. Dodreanu, Pascal Berna, Emmanuel Delay

**Affiliations:** ^a^Departments of Plastic, Reconstructive and Aesthetic Surgery; ^b^Thoracic Surgery, Amiens University Hospital, place Victor Pauchet, Picardie F-80054, Amiens Cedex 1, France; ^c^Department of Plastic Surgery, Leon Bérasd Center, Lyon, France

## Abstract

**Background:** Pectus excavatum is a common congenital deformity involving the anterior thoracic wall. It can be treated with several surgical approaches. **Material and methods:** To our best of knowledge, this is the first case of pectus excavatum repair via a 2-stage double thoracodorsal artery perforator flap procedure in a 37-year-old patient. **Results:** We obtained a satisfactory result in which the missing volume was correctly replaced in the absence of dorsal sequelae. The patient was very satisfied despite the dorsal scars. **Conclusion:** This new approach broadens the surgeon's options for the correction of thoracic deformities.

Pectus excavatum (also called “funnel chest”) is a common congenital deformity involving the anterior thoracic wall. It can be treated with several surgical procedures.^1^ Here, we report an original surgical repair of a mild case of pectus excavatum by using 2 thoracodorsal artery perforator (TDAP) flaps.

## CASE REPORT

A 37-year-old male patient was referred to our department with a stage II pectus excavatum (according to Chin's classification) and a median depression (height = 12 cm; width = 13 cm) involving the sternum and the sternal cartilages. There were no functional complaints.

**Figure 1:**
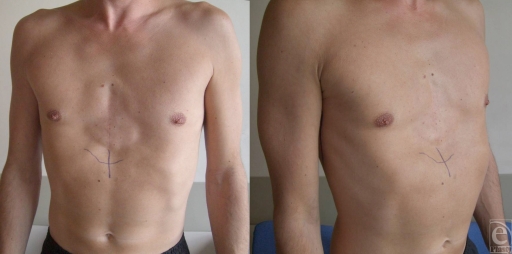
Preoperative views. We can notice not only the median defect but also the prominent lower rib cage.

Three years before, the patient had undergone pectus excavatum repair with a silicone implant. One year after this first operation, contour deformity had been corrected by autologous fat injection (Fig 1).

Despite this treatment, the patient was not satisfied with the result from an aesthetic standpoint, which was associated with a psychological distress. After performing a thorough consultation and informing the patient of his surgical options, we decided to repair the thoracic defect in 2 stages by placing 2 de-epithelized TDAP flaps subcutaneously.

**Figure 2:**
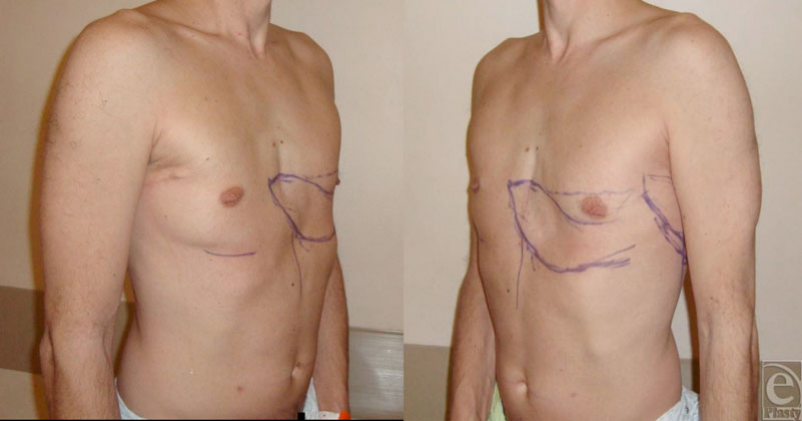
Preoperative views before the second flap. The left flap and the defect are marked with the patient in a standing position.

In the first surgical step, we raised a pedicled TDAP flap on the right side and placed it into the right and lower parts of the defect. Four months later, the left flap was raised and set into the left presternal defect (Fig 2).

The flap harvest procedure was similar on both sides. After locating the first TDAP with color Doppler ultrasonography, we designed an elliptical skin island with a horizontal long axis. The horizontal placement of the flap is a compromise between skin laxity and the scar position. The patient was placed in a lateral position. The border of the flap was incised, and the dissection was carried out from the distal border to the proximal border in the subfascial plane in order to obtain a greater volume as possible. The flap was islanded on the perforator (an intramuscular perforator on both sides) that was dissected down to its origin at the thoracodorsal vessels. The thoracodorsal nerve was preserved.

**Figure 3:**
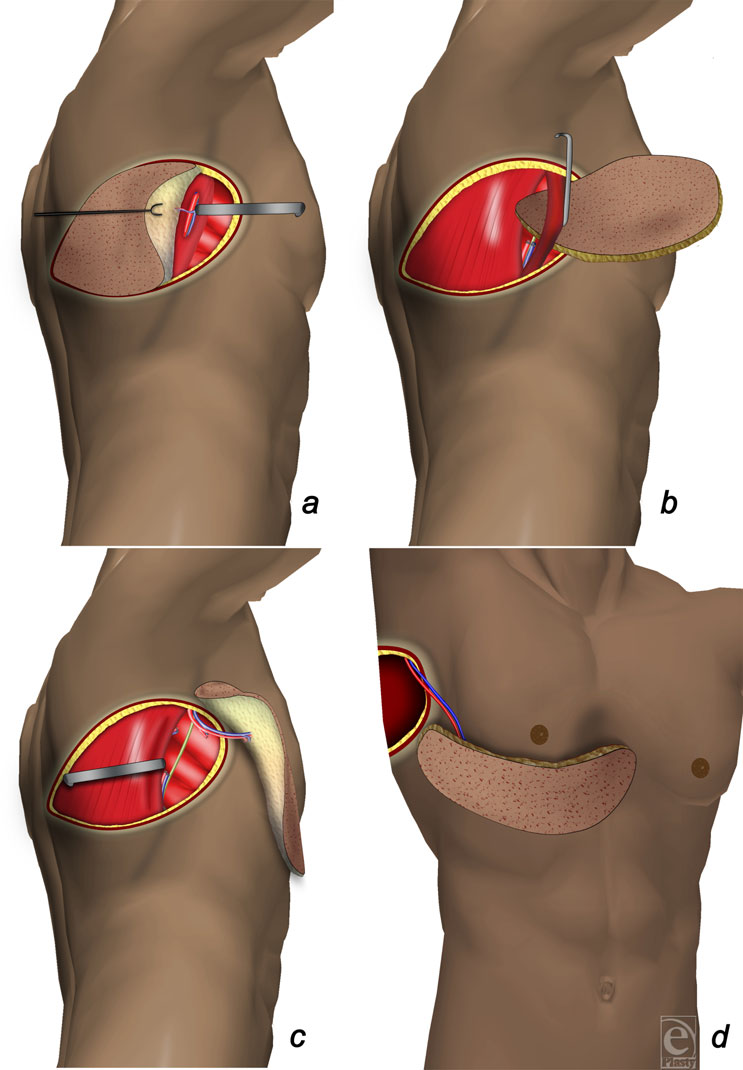
(*a*) First the cutaneous island is de-epithelized and harvested on the perforator vessels. (*b*) The perforator is isolated until the thoracodorsal vessels and then the flap is passed through the dissected muscle. (*c*) The dissection of the thoracodorsal vessel is completed until the axillary vessels to have enough length in order to reach the contralateral chest. The nerve of the latissimus dorsi is preserved. (*d*) The flap is placed in an anterior subcutaneous pocket to fulfill the pectus.

A subcutaneous tunnel was created on the anterior thoracic wall and the flap was placed subcutaneously into the defect (Fig 3).

The donor area was closed (primarily without drainage) in 2 layers with inverted sutures. Undermining the dorsal skin was not necessary; we simply used the skin's natural laxity to close the dorsal defect. For the flap inset, the patient was rolled into the supine position. We incised the previous presternal midline scar (used for implant introduction) and completed the creation of the subcutaneous pocket. The flap was then positioned and fixed in place with transcutaneous sutures over bolsters. The original implant was left in place.

## RESULTS

**Figure 4:**
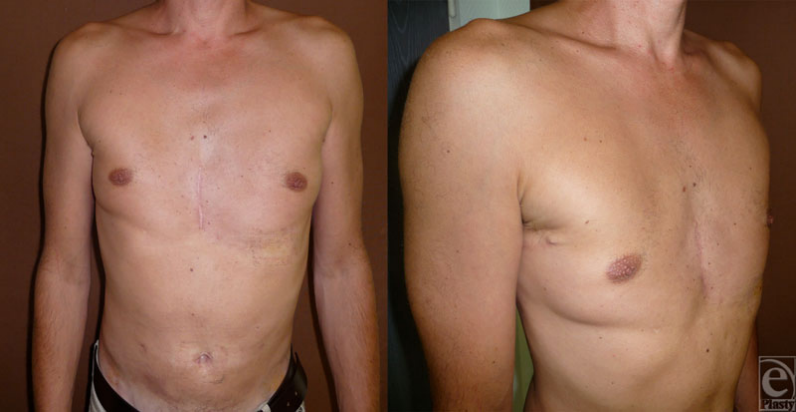
Six months postoperative views.

**Figure 5:**
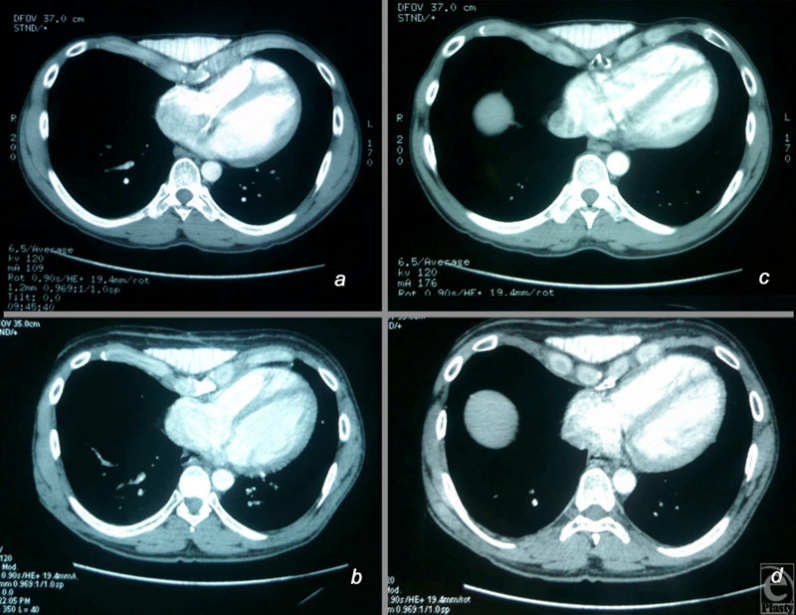
(*a*) and (*c*) Preoperative computed tomographic (CT) scan views showing the thickness over the prothesis before surgery. (*b*) and (*d*) Six months postoperative CT scan views at the same level. On can notice the flaps just over the prothesis.

The patient was released from hospital 2 days after the surgery, with a simple pain medication. The postoperative course was uneventful (no complications were noted), and the patient soon resumed his activities (Fig 4). A computed tomographic scan of the thorax performed 6 months later showed that the missing volume had been correctly restored and had a stable, symmetrical appearance (Fig 5).

The patient was really satisfied with the result despite the donor site scars. The scars did not concern the patient because these were not visible and were on the opposite side of the preoperative anterior deformity.

## DISCUSSION

To the best of our knowledge, this was the first use of a double TDAP flaps for pectus excavatum repair. We chose this approach because the other treatment options (listed in the following text) did not appear to be appropriate for our patient:

1. Replacement of the initial prosthesis with a larger one. This would have increased the risk of complications (seroma, poor position, pain, the edge of the prosthesis being visible through skin, etc).^2^ In fact, the particular shape of this patient's thorax (with a concave lower costal rib) meant that this type of reconstruction was far from ideal.
2. Resection techniques or minimally invasive repair (the Nuss technique). This type of major surgery in a case presenting only aesthetic and psychological complaints without any physiological consequences (the patient's cardiopulmonary function was normal) appeared to us to have an unfavorable risk/-benefit ratio.^3^
3. Multiple free fat transplants.^4^ This patient did not have sufficient or adequate donor sites.

The literature contains few reports of the use of flaps for pectus excavatum repair. Muscle flaps (rectus abdomini flap and latissimus dorsi flap)^5^,^6^ have been used, but donor site morbidity and variable muscle atrophy are major drawbacks in such cases. Finally, repairing pectus excavatum with autologous tissue offers a natural, stable result, as in the case of breast reconstruction.

After the initial description in 1995, several large series^7^,^8^ of TDAP flaps were published, but none were related to pectus excavatum. Our approach offers a precise, stable volume/surface reconstruction, with 2 dorsal scars and no risk of secondary atrophy. The absence of muscle dissection and undermining reduce postoperative pain and the length of hospitalization. It is possible to perform a 1-stage repair of this type of deformity by harvesting bilateral TDAP flaps in a prone position (as it is performed in the case of breast reconstruction.^9^

By capitalizing on the experience gained in mammary reconstruction with a combination of a flap and a prosthesis, a major anterior thoracic deformity could be repaired by using a small implant covered with a perforator flap, thus hiding the implant's edges and obtaining a natural look and feel as a result.^10^

## CONCLUSION

A consensus on pectus excavatum repair has yet to be reached. Here, we describe what we believe to be the first case of pectus excavatum repair by using a double thoracodorsal flap in a young patient. This new approach is worth considering when dealing with patients presenting with pectus excavatum mainly related to aesthetic complaints.
